# Clinical, Neurophysiological and Neuroimaging Findings of Critical Illness Myopathy After COVID-19

**DOI:** 10.7759/cureus.13807

**Published:** 2021-03-10

**Authors:** Ozlem Kayim Yildiz, Bulent Yildiz, Onur Avci, Mursit Hasbek, Sumeyra Kanat

**Affiliations:** 1 Neurology, Cumhuriyet University School of Medicine, Sivas, TUR; 2 Radiology, Cumhuriyet University School of Medicine, Sivas, TUR; 3 Anesthesiology and Reanimation, Cumhuriyet University School of Medicine, Sivas, TUR; 4 Microbiology, Cumhuriyet University School of Medicine, Sivas, TUR

**Keywords:** sars-cov-2, covid-19, critical illness myopathy, muscle mri, case report

## Abstract

Hypoxemic respiratory failure caused by coronavirus disease 2019 (COVID-19) may lead to prolonged intensive care unit stay and mechanical ventilation. Critically ill patients often develop intensive care unit acquired weakness (ICUAW), which is an umbrella term that encompasses critical illness polyneuropathy and critical illness myopathy. The aim of this paper is to report the clinical, neurophysiological, and radiological findings suggesting critical illness myopathy in three patients with critical COVID-19. Muscle magnetic resonance imaging may serve as a diagnostic tool for critical illness myopathy. Weaning failure and generalized muscle weakness with preserved sensation and cranial nerve function should alert physicians for ICUAW.

## Introduction

Coronavirus disease 2019 (COVID-19) is predominantly a respiratory disease; however, neurological manifestations with potential long-term consequences are increasingly being recognized. According to a recent meta-analysis, muscle injury or myalgia is the most common neurologic symptom of COVID-19 [1]. Elevated creatine kinase (CK) levels and rhabdomyolysis have also been reported [2]. However, as neurophysiological studies or other diagnostic tests to detect myopathic changes were not performed in this population to date, the reasons for the elevated CK levels are not clear [1].

Five percent of COVID-19 patients are categorized as ‘critical’ [3]. These patients suffer from respiratory failure, septic shock, and/or multiple organ dysfunction and often require long periods of mechanical ventilation (MV) [3]. Prolonged MV, multiple organ failure, and glucocorticoid use may contribute to intensive care unit (ICU)-acquired weakness (ICUAW) in critical COVID-19 patients. Here, we aim to report the clinical, neurophysiological, and muscle magnetic resonance imaging (MRI) findings of three patients who developed ICUAW after prolonged MV and ICU stay for critical COVID-19.

## Case presentation

A 64-year-old woman was admitted to the hospital because of fever and cough. Computed tomographic images of the lungs showed ground-glass opacities suggesting COVID-19 pneumonia. The diagnosis was confirmed by nasopharyngeal swab testing for severe acute respiratory syndrome coronavirus 2 (SARS-CoV-2) ribonucleic acid (RNA). Shortly after onset, the patient was transferred to the ICU and underwent endotracheal intubation and MV. She received convalescent plasma, tocilizumab, and dexamethasone during her ICU stay. She was not given neuromuscular blocking agents.

Ninety days after symptom onset, a neurological examination, performed for weaning failure, revealed diffuse muscle weakness and atrophy with preserved sensation and cranial nerve functions. Deep tendon reflexes were absent. MRI findings of the brain and the spinal cord and the results of the cerebrospinal fluid analyses were normal. Her serum CK levels were within normal limits.

The patient underwent a neurophysiological examination, including nerve conduction studies (NCSs) and concentric needle electromyography (EMG). The neurophysiological study was performed using Cadwell Sierra Summit electromyograph (Cadwell Laboratories, Kennewick, WA, USA). The findings are summarised in Table 1. Compound muscle action potential (CMAP) amplitudes were reduced with normal distal motor latencies (DML) and conduction velocities (CV). CMAPs were prolonged and smoothly outlined. The sensory NCSs were within normal limits. No decremental response was observed with 3 Hz, 5 Hz, and 10 Hz repetitive nerve stimulation (RNS). Needle EMG showed fibrillation potentials and positive sharp waves in some muscles. Brief, small, and polyphasic motor unit action potentials (MUAPs) with normal or early recruitment pattern were recorded.

**Table 1:** Neurophysiological findings CMAP, compound muscle action potential; SNAP, sensory nerve action potential; F. P. and PSWs, fibrillation potentials and positive sharp waves; MUAP, motor unit action potential; NCS, nerve conduction studies; EMG, electromyography; EDC, extensor digitorum communis; FDI, first dorsal interosseous; APB, abductor pollicis brevis; TA, tibialis anterior; EDB, extensor digitorum brevis.

	Distal latency (ms)	CMAP amplitude (mV)	Motor conduction velocity (m/s)	SNAP amplitude (µV)	Sensory conduction velocity (m/s)	F.P. and PSWs	MUAPs
NCS							
Median, L	4.5	1.1	63	13	49		
Ulnar, L	3.9	0.5	49	9	50		
Peroneal, L	4.4	0.1	52				
Tibial, L Fossa stimulation (Recording on gastrocnemius muscle)	6.1	0.1	49				
Sural, L	2.5	12		46			
EMG							
Deltoid, L						+	Short-duration and low amplitude, early interference
Biceps brachii, L						+	Short-duration and low amplitude, early interference
EDC, L						+	Short-duration and low amplitude, normal interference
FDI, L						-	Short-duration and low amplitude, early interference
APB						-	Short-duration and low amplitude, early interference
TA						+	Short-duration and low amplitude, early interference
EDB						-	Short-duration and low amplitude, normal interference

**Figure 1:**
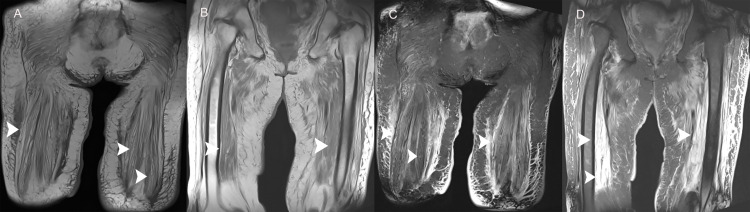
Coronal T1-weighted and coronal fat suppressed T2-weighted images of the pelvis and the thighs. Coronal T1-weighted images of the pelvis (a) and the thigh (b) show diffuse muscle atrophy. Coronal fat suppressed T2-weighted images of the pelvis (c) and the thighs (d) show diffuse muscle atrophy and hyperintense signal changes indicating muscle edema and inflammation (arrowheads).

Coronal T1-weighted and fat-suppressed T2-weighted MRI of the thighs and the pelvis demonstrated signals consistent with diffuse muscle atrophy and edema (Figure 1).

A diagnosis of critical illness myopathy (CIM) was made and the patient could not be liberated from mechanical ventilation despite an intensive rehabilitation program. She died because of sepsis and multi-organ failure four months later.

**Figure 2:**
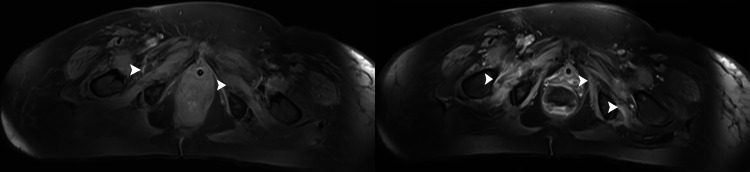
Axial fat-suppressed T1-weighted images of the pelvis. The images show (a) muscle atrophy and (b) gadolinium-enhancement (arrowheads).

We also studied two other patients with COVID-19 who developed weaning failure and tetraparesis after prolonged MV and ICU stay (patient numbers 2 and 3, Table 2). They had been treated with corticosteroids and sedatives. The patients’ neurophysiological findings were suggestive of CIM with significantly reduced CMAP amplitudes, normal DMLs, CVs, sensory NCSs, and RNS studies, and short duration and low amplitude MUAPs with early recruitment pattern. Diffuse muscle atrophy, edema, and gadolinium-enhancement in the pelvic muscles were detected on MRI (Figure 2). Patient 2 died after 61 days of hospitalization and patient 3 was liberated from MV after 57 days. She is recovering slowly with intensive physiotherapy.

**Table 2:** The demographic characteristics and the outcomes of the patients F, female; ICU, intensive care unit.

Patient	Age	Gender	Time in ICU	Outcome
1	64	F	>200 days	Deceased
2	76	F	60 days	Deceased
3	81	F	61 days	Liberated

## Discussion

ICUAW refers to muscle weakness acquired during critical illness. ICUAW originating from a myopathic disturbance is labeled CIM. Neurophysiological studies and muscle biopsy should be performed for diagnosis [4]. CMAPs are of low amplitudes and DMLs and nerve CVs are within normal limits. Markedly prolonged CMAPs are typical. Sensory NCSs are normal. On needle EMG, polyphasic, short duration and low amplitude MUAPs, early or normal recruitment and occasional fibrillation potentials and positive sharp waves are typical findings. RNS should be performed to rule out a neuromuscular transmission deficit. Muscle MRI may be a supportive diagnostic tool. Typical findings, including increased T2/STIR signal and gadolinium enhancement on T1, have been reported to correlate with muscle biopsy findings suggesting CIM [5].

Two cases with CIM after prolonged ICU stay for critical COVID-19 have been reported to date [6,7]. In these cases, the diagnosis was made using clinical and neurophysiological findings. To the best of our knowledge, muscle MRI findings of CIM patients surviving from critical COVID-19 have not been reported to date.

## Conclusions

In conclusion, COVID-19 patients requiring prolonged ICU stay and MV may be at risk for ICUAW with devastating short- and long-term consequences. Muscle MRI could serve as a supportive diagnostic tool. Further studies are warranted to determine the incidence of ICUAW in COVID-19 survivors.
